# Understanding the potential contribution of polygenic risk scores to the prediction of gestational and type 2 diabetes in women from British Pakistani and Bangladeshi groups: a cohort study in Genes and Health

**DOI:** 10.1016/j.xagr.2025.100457

**Published:** 2025-02-21

**Authors:** Julia Zöllner, Binur Orazumbekova, Sam Hodgson, David A. van Heel, Genes and Health Research Team, Shaheen Akhtar, Mohammad Anwar, Omar Asgar, Samina Ashraf, Saeed Bidi, Gerome Breen, Eamonn Maher, Daniel MacArthur, Dan Mason, Bill Newman, Caroline Winckley, John Wright, James Broster, Raymond Chung, David Collier, Charles J Curtis, Shabana Chaudhary, Grainne Colligan, Panos Deloukas, Ceri Durham, Faiza Durrani, Fabiola Eto, Joseph Gafton, Ana Angel, Chris Griffiths, Joanne Henry, Teng Heng, Qin Qin Huan, Matt Hurles, Karen A Hunt, Shapna Hussain, Kamrul Islam, Vivek Iyer, Benjamin M Jacobs, Georgios Kalantzis, Ahsan Khan, Claudia Langenberg, Cath Lavery, Sang Hyuck Lee, Sidra Malik, Daniel Malawsky, Hilary Martin, Rohini Mathur, Mohammed Bodrul Mazid, John McDermott, Caroline Morton, Vladimir Ovchinnikov, Elizabeth Owor, Iaroslav Popov, Asma Qureshi, Mehru Raza, Jessry Russell, Nishat Safa, Miriam Samuel, Michael Simpson, John Solly, Marie Spreckley, Daniel Stow, Michael Taylor, Richard C Trembath, Karen Tricker, Klaudia Walter, Suzanne Wood, Sabina Yasmin, Ishevanhu Zengeya, Stamatina Iliodromiti, Moneeza Siddiqui, Rohini Mathur, Sarah Finer, Jennifer Jardine

**Affiliations:** 4 Wellcome Sanger Institute, London, UK (Akhtar, Heng, Huan, Hurles, Iyer, Kalantzis, Malawsky, Martin, and Walter); 5 Social Action for Health, London, UK (Anwar, Colligan, and Durham); 6 Manchester University NHS Trust, Manchester, UK (Asgar); 7 Bradford Teaching Hospitals, Bradford, UK (Ashraf); 8 Queen Mary University of London, London, UK (Bidi, Broster, Collier, Chaudhary, Deloukas, Durrani, Eto, Gafton, Angel, Griffiths, Henry, Hunt, Hussain, Islam, Jacobs, Khan, Langenberg, Lavery, Malik, Mathur, Bodrul Mazid, Morton, Ovchinnikov, Owor, Popov, Qureshi, Raza, Russell, Safa, Samuel, Solly, Spreckley, Stow, Taylor, Tricker, Wood, Yasmin, and Zengeya); 9 King's College London, London, UK (Breen, Chung, Curtis, Hyuck Lee, Simpson, and Trembath); 10 University of Cambridge, Cambridge, UK (Maher); 11 Garvan Institute of Medical Research, Darlinghurst, NSW, Australia (MacArthur); 12 Born in Bradford, Bradford, UK (Mason); 13 University of Manchester, Manchester, UK (Newman and McDermott); 14 NIHR Clinical Research Clinical Trials, Manchester, UK (Winckley); 15 Bradford Institute for Health Research, Bradford, UK (Wright); 1 Institute for Women's Health, Population Health Sciences, University College London, London, UK (Zöllner); 2 Centre for Cell Biology and Cutaneous Research, Blizard Institute, Barts and the London School of Medicine and Dentistry, Queen Mary University of London, London, UK (van Heel); 3 Wolfson Institute of Population Health, Barts and the London School of Medicine and Dentistry, Queen Mary University of London, London, UK (Zöllner, Orazumbekova, Hodgson, Iliodromiti, Siddiqui, Mathur, Finer, and Jardine)

**Keywords:** gestational diabetes, polygenic risk, prediction model, pregnancy complications, prognosis, risk stratification, South Asian

## Abstract

**Background:**

British Pakistani and Bangladeshi (BPB) women have disproportionately high rates of gestational diabetes mellitus (GDM), with prevalence estimates up to three times higher than in the general population. They are also at increased risk of progressing to type 2 diabetes, leading to significant health complications. Despite this, predictive models tailored to this high-risk, yet understudied group are lacking.

**Objective:**

To investigate whether combining genetic and traditional clinical data improves risk prediction of GDM and progression to type 2 diabetes among BPB women. We hypothesized that incorporating polygenic risk scores (PRS) would enhance the predictive accuracy of existing models.

**Study Design:**

An observational cohort study utilizing the Genes & Health dataset, which includes comprehensive electronic health records. Women who gave birth between 2000 and 2023, both with and without a history of GDM, were included. Controls were defined as women without a GDM diagnosis during this period but who had a birth record. A total of 117 type 2 diabetes or GDM PRS were tested to determine the optimal PRS based on predictive performance metrics. The best-performing PRS was integrated with clinical variables for statistical analyses, including descriptive statistics, chi-square tests, logistic regression, and receiver operating characteristic curve analysis.

**Results:**

Of 13,489 women with birth records, 10,931 were included in the analysis, with 29.3% developing GDM. Women with GDM were older (mean age 31.7 years, *P*<.001) and had a higher BMI (mean 28.4 kg/m^2^, *P*<.001) compared to controls. The optimal PRS demonstrated a strong association with GDM risk; women in the highest PRS decile had significantly increased odds of developing GDM (OR 5.66, 95% CI [4.59, 7.01], *P*=3.62×10^−58^). Furthermore, the risk of converting from GDM to type 2 diabetes was 30% in the highest PRS decile, compared to 19% among all GDM cases and 11% in the lowest decile. Incorporating genetic risk factors with clinical data improved the C-statistic for predicting type 2 diabetes following GDM from 0.62 to 0.67 (*P*=4.58×10^−6^), indicating better model discrimination.

**Conclusion:**

The integration of genetic assessment with traditional clinical factors significantly enhances risk prediction for BPB women at high risk of developing type 2 diabetes after GDM. These findings support the implementation of targeted interventions and personalized monitoring strategies in this high-risk population. Future research should focus on validating these predictive models in external cohorts and exploring their integration into clinical practice to improve health outcomes.

**AJOG MFM at a Glance**

**Why was this study conducted?**

To understand the contribution of polygenic risk scores in predicting gestational diabetes and subsequent type 2 diabetes in women of British Pakistani and Bangladeshi descent.

**Key findings**

Women with higher genetic risk scores were found to have a significantly increased likelihood of developing gestational diabetes and type 2 diabetes.

**What does this add to what is known?**

This research enhances understanding of how genetic factors can improve risk prediction in high-risk populations, potentially informing targeted preventive strategies.

## Introduction

Gestational diabetes, or diabetes which newly arises in pregnancy, is a substantial risk factor for type 2 diabetes, with women who have gestational diabetes having at least a seven-fold increase of type 2 diabetes compared to women who have a pregnancy without gestational diabetes.^1^^,^^2^ Understanding the relationship between gestational and type 2 diabetes not only provides important insights into each disease but also opportunities to develop novel interventions to reduce the risk of progression to type 2 diabetes.

This risk of progression is particularly high in women of South Asian ethnic origin, of whom approximately 35% will have impaired glucose tolerance at 6 to 8 weeks postpartum.^3^ People of South Asian ethnic origin are at elevated risk overall of type 2 diabetes, which occurs younger, at a lower BMI and with fewer comorbidities than in Caucasian populations.^4^ Targeting this population is, therefore, a high priority to reduce the overall incidence of type 2 diabetes.

Recent studies have demonstrated that incorporating genetic as well as demographic and disease information can improve the accuracy of prediction of likelihood of type 2 diabetes in women with gestational diabetes mellitus (GDM), including those of South Asian ethnic origin.^5^ Many of the genetic and environmental risk factors associated with GDM are shared with type 2 diabetes.^6^ Women diagnosed with GDM often have a higher likelihood of having at least one parent with type 2 diabetes compared to those with normal gestational glycaemia^7^ and polygenic risk scores (PRS) for type 2 diabetes demonstrate correlation with the risk of developing GDM.^8^^,^^9^ PRSs quantify an individual's inherited risk of disease by summing the weighted contribution of multiple genetic variants identified through genome-wide association studies. PRSs predict the likelihood of developing disorders and are a valuable tool in precision medicine.

Pregnancy offers an opportunity to combine PRS with non-genetic risk factors, allow an early risk assessment to offer preventive interventions and/or medicines. To date only a few small studies have investigated polygenic risk in GDM demonstrating linear correlation between those in the highest risk deciles and glucose intolerance.^10^^,^^11^ The use of PRS provides an opportunity to stratify women more precisely, identifying those at the highest risk who may benefit from targeted counseling and interventions postpartum. This is particularly important as studies like ELOPE study have shown that many women with GDM are unaware of their heightened risk for type 2 diabetes, and current clinical follow-up often fails to provide clear guidance. Incorporating PRS into existing risk assessments could enhance communication, provide clarity about individual risk, and support more personalized postpartum care. No studies have integrated a polygenic risk combined with pregnancy risk factors in a large real-world dataset like Genes and Health to improve clinical prediction. In this study, we aim to understand the demographic, disease, and genetic associations for gestational diabetes and subsequent development of type 2 diabetes among women of South Asian ethnic origin.

## Materials and methods

This is an observational cohort study using the Genes & Health (G&H) cohort of people of British Pakistani and Bangladeshi (BPB) ethnic origin. The population of interest are women who have had a recorded registerable birth between 2000 and 2023 with a history of gestational diabetes.

### Data source and study population

G&H is a long-term research project examining the relationship between Genes and Health in British Bangladeshi and Pakistani communities, which have higher rates of diabetes and other health issues. By linking genetic data with health records, the study aims to identify genetic factors that contribute to disease and improve understanding of disease risk in these communities and the general population. The study excludes Indian subgroups due to the focus of other projects, such as the LOLIPOP study (www.sabiobank.org). Previous findings from our group suggest differences in the genetic burden of T2D and GDM between these groups, underscoring the potential for variation across South Asian subpopulations.^12^ Genes and Health recruits BPB people aged 16 years and above, predominantly from community and primary care settings.^6^ We used the 2023 data release (secondary care data May 2023, primary care data November 2023), which comprised electronic health record (EHR) data from primary and secondary care, and genotype data from the Illumina Infinium Global Screening Array V3 Chip^13^ Descriptions of quality control, imputation of genotype data, as well as filtering and principal component analysis are provided in Supplementary Methods.

Women from the G&H population were included if they had a SNOMED or ICD-10 code (Supplementary Table 1) indicating that they had given birth on or after April 1, 2000, and on or before the latest EHR data refresh.^14^ Births prior to this year were excluded a priori as the quality of birth records in electronic health data in England improved from 2000.

### Determination of exposures and outcomes

For this study, the primary exposure was gestational diabetes, and the primary outcome was development of type 2 diabetes following pregnancy. We identified women as having gestational diabetes if they had a code for gestational diabetes (Supplementary Table 1) within the 6 months preceding or 6 weeks following a birth episode in either HES or the GP record from the year 2000 onwards. We additionally inspected women with a code of type 2 diabetes in this period. If diabetes was only coded in pregnancy and the postpartum period up to 3 months, and only treated with metformin and insulin (as recommended by the National Institute for Clinical Excellence^15^) then this was considered to indicate a diagnosis of gestational diabetes. If diabetes was coded or treatment prescribed more than 6 months before birth, or after 3 months postpartum, and/or if suphonylureas were prescribed, then the record was treated as indicating diabetes outside of pregnancy.

The following additional filtering approaches were applied for mothers with multiple pregnancy records in order to keep a single pregnancy per mother for analysis: (1) if GDM was not diagnosed at any of the pregnancies, phenotype data at the latest available time point was kept (ie, keep older women without GDM), (2) if GDM was diagnosed during any of the pregnancies included in the study, the earliest time point where GDM was diagnosed was kept (ie, keep younger women with GDM). When assessing parity—we used GDM diagnosis dates and birth record dates to assess which came first. Due to higher rates of relatedness in this specific population, related individuals by third degree or closer were removed from the analysis using kinship coefficients from KING v2.3.4.^16^

Covariates included age at time of giving birth, BMI closest to or during pregnancy, parity (nulliparous or multiparous), and ethnicity (Bangladeshi or Pakistani). These were used to derive an epidemiological model.

### Choice of PRS

At the time of the analysis there were no South Asian-specific type 2 diabetes PRS data available, therefore type 2 diabetes (*n*=115) and GDM (*n*=2) PRS available on the PGS catalog were compared against performance in our dataset (Supplementary Figure 1). In addition, weights were derived from the DIAGRAM's 2022 multi-ethnic type 2 diabetes GWAS meta-analysis, which included 8.3% South Asians (51.1% European, 26.4% East Asian, 6.6% African ancestry and 5.6% other ethnic backgrounds).^17^ Finally, weights were derived from the largest and most recent GDM GWAS (100% European—Finland and Estonia).^18^ PGS002771 (100% European) performed the best and was used for subsequent analysis; the derivation of this PRS is described elsewhere.^19^

### Statistical analysis

Participant characteristics were evaluated using descriptive statistical methods. For continuous variables that followed a normal distribution, the mean and standard deviation are presented. For variables that were not normally distributed, the median and interquartile range (IQR) are also included. Categorical variables are summarized as proportions. A two-sided p-value of less than 0.05 was considered statistically significant. Data analysis was performed on the Google Cloud Trusted Research environment. For statistical analysis, RStudio version 2023.09.1 was used.

### Missing data

Missing data was imputed using the R program Mice. We imputed BMI values for 1010 volunteers (763 controls, 247 cases) and parity for 394 cases using multiple imputation with m=10. Estimates were pooled using Rubin's rules.^17^

### Ethical approval

ELGH (East London Genes and Health) operates under ethical approval, 14/LO/1240, from London South East NRES Committee of the Health Research Authority, dated 16 September 2014. This protocol was approved by ELGH on July 5, 2023, reference S00099.

## Results

The Genes and Health dataset includes 59,150 participants, of whom 32,494 are recorded as women. Of these women, 13,489 had a record of giving birth after January 1, 2000, in either their primary or secondary care record; 27,681 discrete birth episodes were identified. Women who had a birth record were generally younger (controls median year of birth 1982 [IQR 1977–1987]; cases mean 1983 [IQR 1979–1987]) compared to participants who had no record of having given birth (median 1979 [IQR 1967–1990]. Median number of births in the cohort was two per woman.

**Figure 1:**
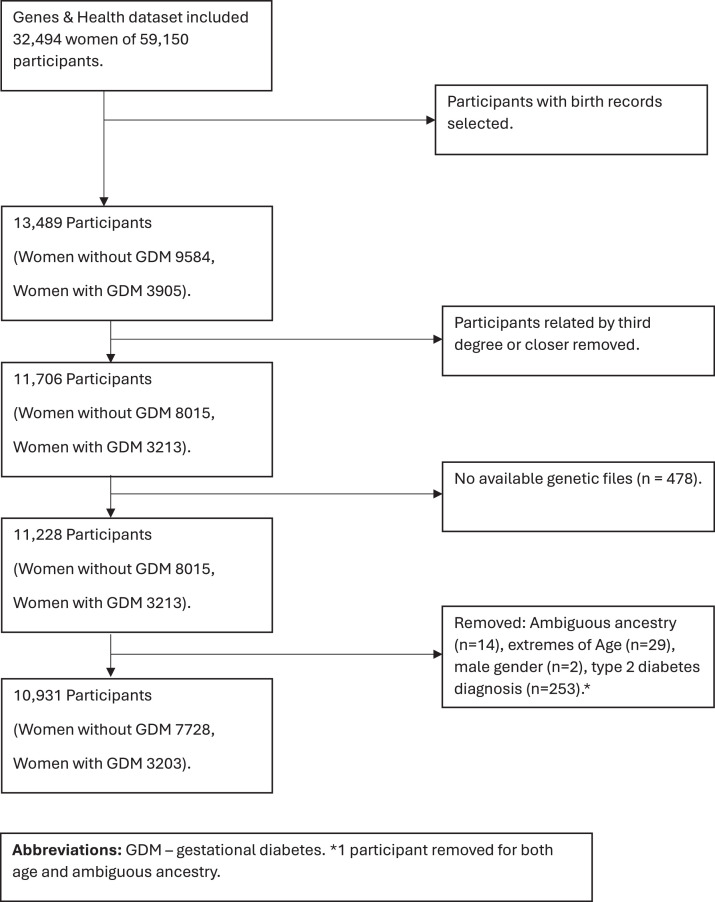
Strobe chart

10931 women were included in the analysis (Figure 1). The demographics of the included cases and controls are described in Table 1. 29.3% of women in the cohort developed GDM in their included pregnancy. Women who developed GDM were slightly older (mean age 31.7 compared to 31.3 years, *P*<.001) and had a higher BMI (mean BMI 28.4 compared to 27.3 kg/m^2^, *P*<.001), and more likely to be Bangladeshi than women who did not have GDM (75.3% of all women with GDM were Bangladeshi compared to 59.7% of all women without GDM, *P*<.001). Nulliparous women were more likely to be represented in the GDM group (52.7% compared to 29.5%, *P*<.01).

Zöllner. Understanding the potential contribution of polygenic risk scores to the prediction of gestational and type 2 diabetes in women from British Pakistani and Bangladeshi groups. Am J Obstet Gynecol MFM 2025.

**Table 1:** Characteristics of 10,931 Genes and Health participants who gave birth after April 1, 2000

Characteristic	Women who did not have GDM (*n*, %)	Women who had GDM (*n*, %)	*P* value
All women	7728 (70.70%)	3203 (29.30%)	.
Age (y), mean (SD)	31.36 (4.92)	31.73 (5.24)	<.001
BMI, mean (SD)^a^	27.33 (5.14)	28.47 (5.19)	<.001
Parity at included birth, *n* (%)^b^			
Nulliparous	2059 (29.58)	1373 (52.75)	<.001
Multiparous (1 or more)	4902 (70.42)	1230 (47.25)	
Genetic ancestry, *n* (%)			
British Bangladeshi	4611 (59.7)	2412 (75.3)	<.001
British Pakistani	3117 (40.3)	791 (24.7)	
Polygenic risk score for T2DM, *n* (%)			
1st decile	949 (12.28)	145 (4.53)	<.001
10th Decile	586 (7.58)	507 (15.83)	

Characteristics of participants (cases and non-cases) included in the analysis. Data is presented as means (standard deviation) unless otherwise stated. P-values are calculated from Chi-squared test for categorical variables and independent *t* test for continuous variables. GDM diagnosis is made based on coding in primary and secondary care records.

GDM, gestational diabetes; SD, standard deviation; T2DM, type 2 diabetes.

a There are 9.2% missing BMI entries (*n*=1010)

b There are 3.6% missing Parity entries (*n*=394).

### Performance of PRS in predicting GDM

**Figure 2:**
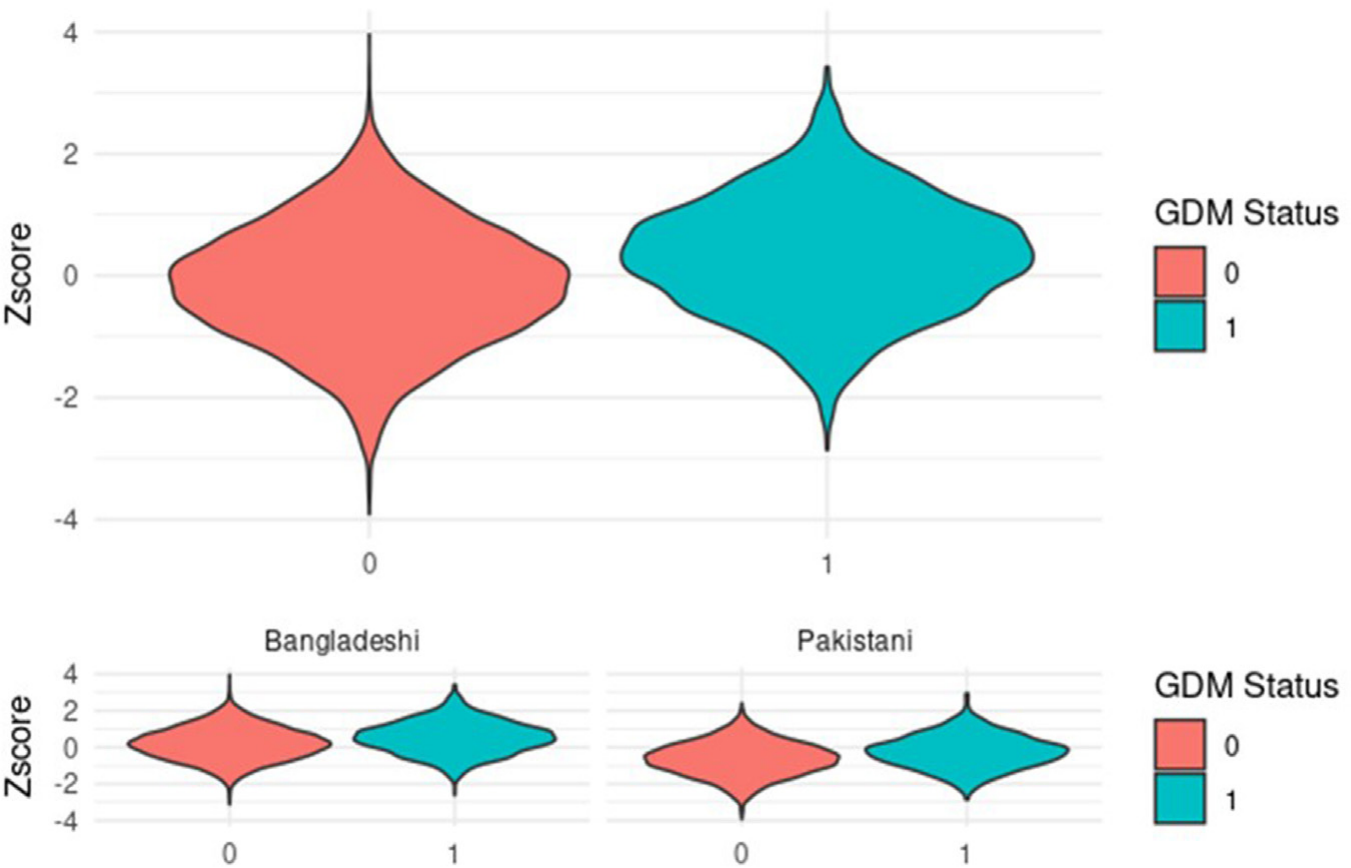
Polygenic risk score distribution amongst women with and without GDM GDM status: 0=women who did not have GDM; 1=women who had GDM. *GDM*, gestational diabetes

The performance of the PRS in predicting GDM is shown in Figure 2. The PRS *z*-score was higher in women with GDM compared with controls (mean *z*-score GDM 0.34 ± 0.95 vs controls –0.14 ± 0.99; *P*<2.2×10⁻¹⁶). British Bangladeshi women (mean *z*-score GDM 0.19 ±0.89 vs controls 0.52 ±0.89; *P*=2.2×10^−16^) had higher *z*-scores compared to Pakistani women (mean *z*-score GDM –0.24 ±0.89 vs Controls –0.63 ±0.92; *P*=2.2×10^−16^). A histogram depicting the distribution of the PRS across the two groups can be found in (Supplementary Figure 2).

Zöllner. Understanding the potential contribution of polygenic risk scores to the prediction of gestational and type 2 diabetes in women from British Pakistani and Bangladeshi groups. Am J Obstet Gynecol MFM 2025.

### Association between PRS and risk of developing GDM

**Figure 3:**
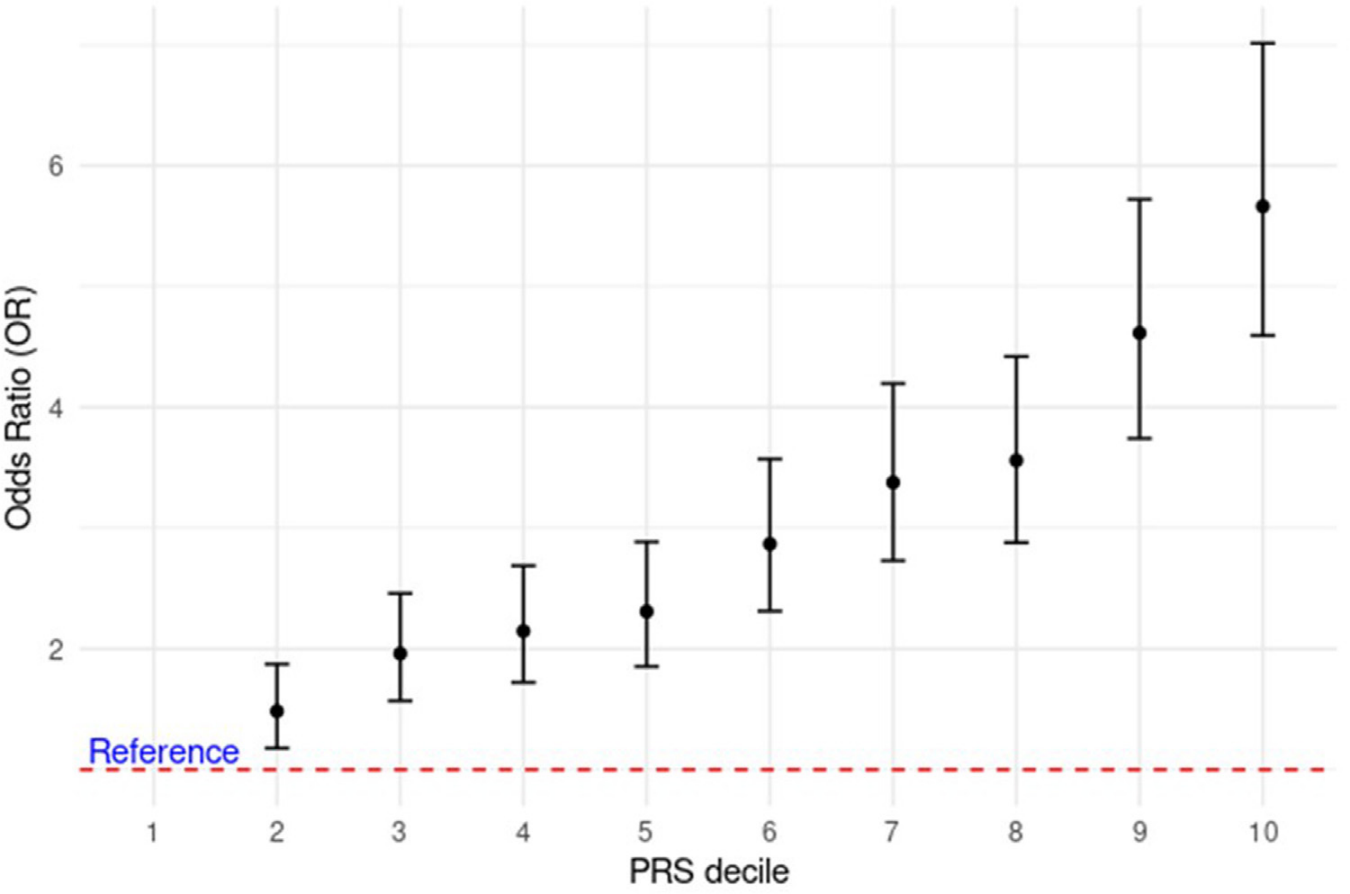
Risk of developing gestational diabetes by polygenic risk score decile *PRS*, polygenic risk score.

To further investigate the relationship between the PRS and GDM risk, we divided our participants into deciles based on their PRS values. As demonstrated in Figure 3, the likelihood of diabetes increased incrementally with increasing PRS deciles. The odds of having gestational diabetes in the 10th decile compared to the 1st decile were 5.66 (95% CI=[4.59, 7.01], *P*=3.62 × 10^–58^). These results were replicated in the subcohorts with British Bangladeshi women in the highest risk decile being 3.8 (95% CI=[3.01, 4.82], *P*=1.14 × 10^–34^) and Pakistani women in the highest risk decile 5.03 (95% CI=[3.42, 7.54], *P*=9.14 × 10^–15^) more likely to develop GDM, compared to those in the lowest risk decile (Supplementary Figure 3).

Zöllner. Understanding the potential contribution of polygenic risk scores to the prediction of gestational and type 2 diabetes in women from British Pakistani and Bangladeshi groups. Am J Obstet Gynecol MFM 2025.

### Comparison of null (population risk factors only), genetic, and combined models in predicting gestational diabetes using AUC

**Figure 4:**
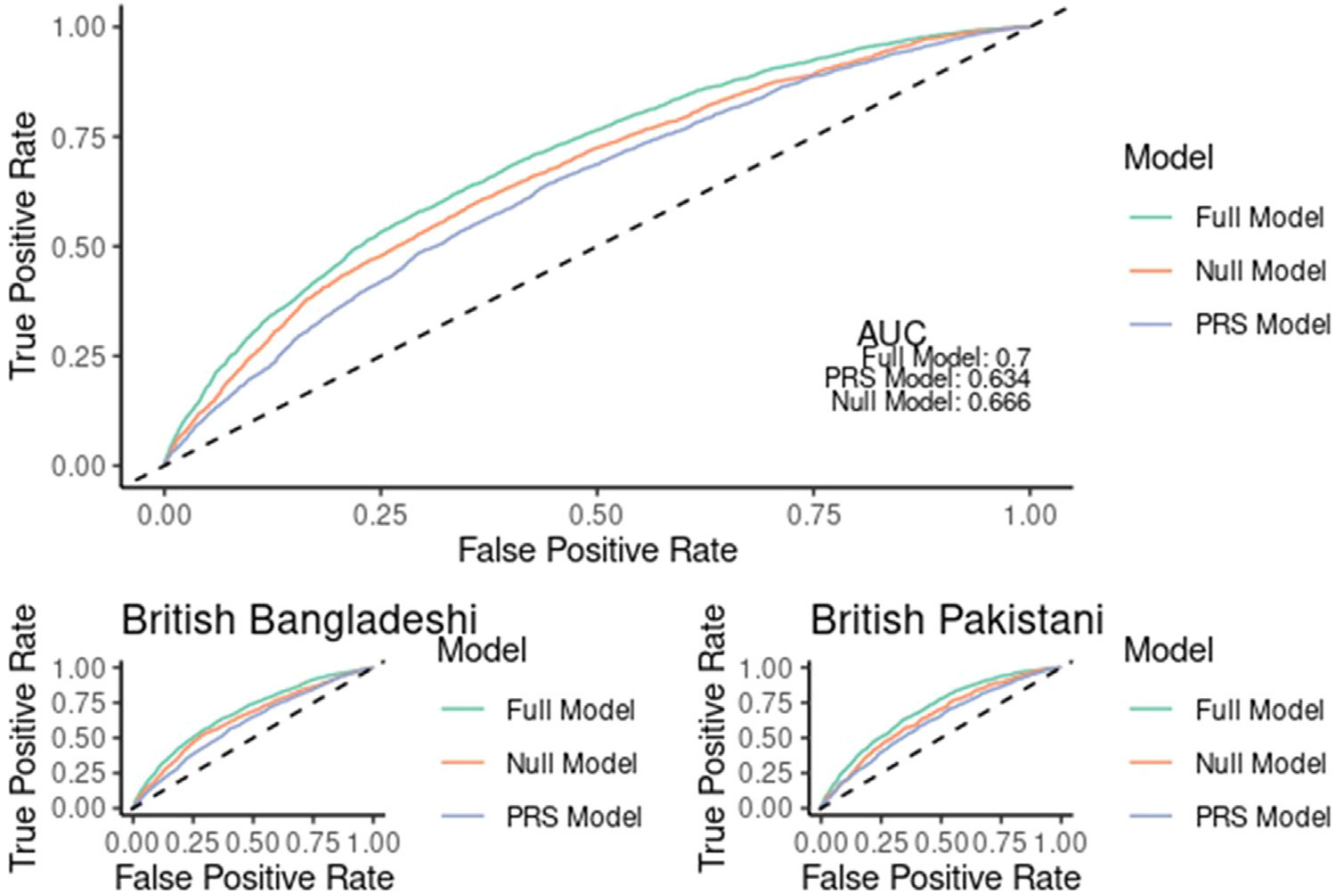
ROC curves demonstrating improvement in prediction of GDM with utilization of genetic information Comparing three models: the Null model, which includes only traditional population risk factors (age, BMI, parity, and ethnicity); the PRS model, which includes only the polygenic risk score; and the Full model, which combines both the population risk factors and the polygenic risk score. *AUC*, area under the curve; *PRS*, polygenic risk score; *ROC*, receiver operating characteristic.

The receiver operating curve (ROC; Figure 4) demonstrates that combining population and genetic risk improves comparison of gestational diabetes when compared to the null model using population risk factors only. There is an improvement in the C-statistic from 0.66 (95% CI=[0.65, 0.67]) to 0.70 (95% CI=[0.68, 0.71]); this improvement is statistically significant with a p-value of < 2.2×10⁻¹⁶. These results were also replicated in stratified subcohorts of British Bangladeshi (Full model AUC=0.68, 95% CI=[0.66–0.69] vs null model AUC=0.64, 95% CI=[0.62–0.65]), *P*=2.2×10⁻¹⁶ and Pakistani women (Full model AUC=0.69, 95% CI=[0.67–0.71] vs null model AUC=0.65, 95% CI=[0.62–0.67], *P*=0.14 × 10^–8^).

Zöllner. Understanding the potential contribution of polygenic risk scores to the prediction of gestational and type 2 diabetes in women from British Pakistani and Bangladeshi groups. Am J Obstet Gynecol MFM 2025.

### Performance of PRS in predicting transfer from GDM to type 2 diabetes

**Figure 5:**
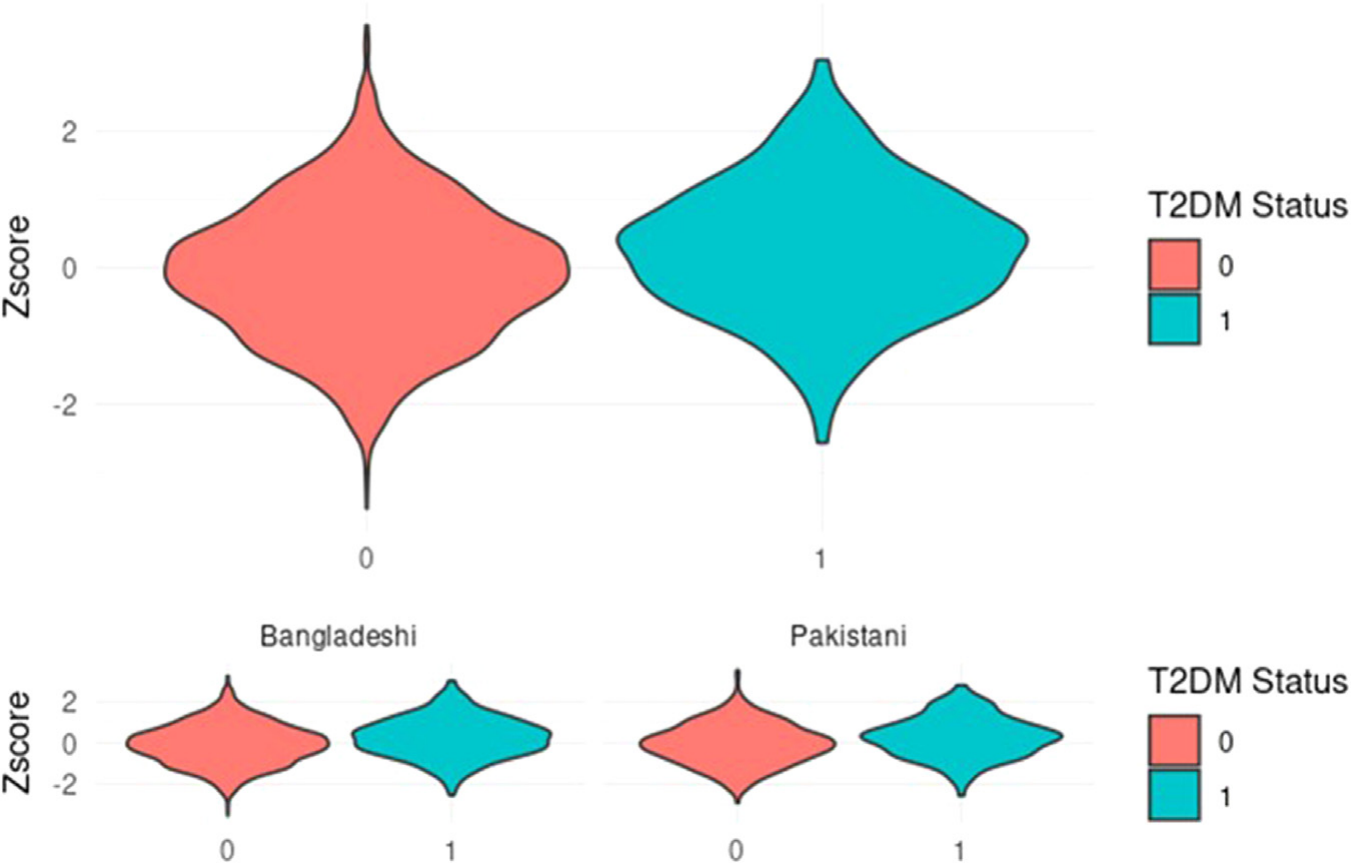
Polygenic risk score distribution amongst women with GDM who progressed to type 2 diabetes versus those who did not T2DM status: 0=women who did progress to type 2 diabetes; 1=women who progressed to type 2 diabetes. *T2DM*, type 2 diabetes

The performance of the PRS in predicting development of type 2 diabetes following GDM is shown in Figure 5. The PRS *z*-score was higher in women with GDM who progressed to type 2 diabetes (T2DM) compared with those that did not go on to develop T2DM (mean *z*-score GDM to T2DM 0.27 ± 0.97 vs no progression to T2DM –0.06 ± 0.99; *P*<6.704×10⁻^14^). British Bangladeshi women (mean *z*-score GDM to T2DM 0.27 ±0.97 vs no progression to T2DM –0.06 ±0.99; *P*=4.169 × 10^–11^) had lower *z*-scores compared to Pakistani women (mean *z*-score GDM toT2DM 0.28 ±0.98 vs no progression to T2DM –0.05 ±0.99; *P*=.00026). Individuals with a previous history of gestational diabetes in the highest decile were 3.44 times more likely to develop type 2 diabetes.

Zöllner. Understanding the potential contribution of polygenic risk scores to the prediction of gestational and type 2 diabetes in women from British Pakistani and Bangladeshi groups. Am J Obstet Gynecol MFM 2025.

Over 10 years, there was no significant difference between days to progression to type 2 diabetes between the first and highest decile (median days highest decile 1213 [IQR: 646–2073] compared to lowest decile 1562 [IQR: 828–2863], *P*=.141; Supplementary Figure 4). There was no evidence of younger age at the time of type 2 diabetes onset between the highest and lowest decile (Supplementary Figure 5). Table 2 demonstrates the percentage prediction of risk of conversion from gestational diabetes to type 2 diabetes.

**Table 2:** Percentage prediction of risk of conversion from gestational diabetes to type 2 diabetes, based on combined model incorporating polygenic risk score and population risk factors (age, parity, BMI)

Prediction of risk	Conversion to type 2 diabetes
Lowest decile of predicted risk	11%
All GDM cases	19%
Highest decile of predicted risk	30%

GDM, gestational diabetes.

### Comparison of null (population risk factors only), genetic, and combined models in predicting development of type 2 diabetes following gestational diabetes using AUC

**Figure 6:**
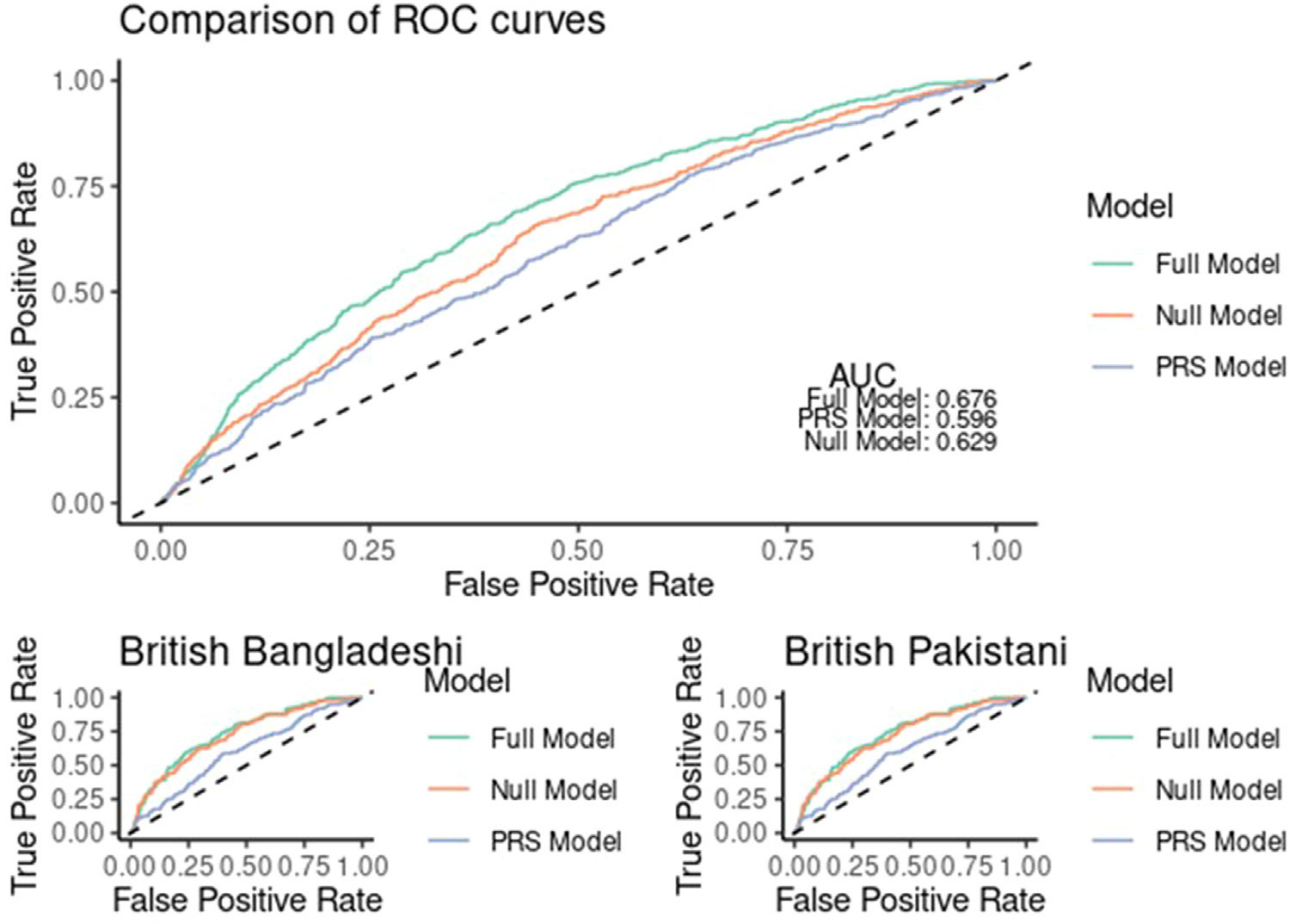
ROC curves demonstrating improvement in prediction of type 2 diabetes with utilization of genetic information Comparing three models: the Null model, which includes only traditional population risk factors (age, BMI, parity, and ethnicity); the PRS model, which includes only the polygenic risk score; and the Full model, which combines both the population risk factors and the polygenic risk score. *AUC*, area under the curve; *PRS*, polygenic risk score; *ROC*, receiver operating characteristic.

The ROC (Figure 6) demonstrates an improvement in performance by addition of genetic risk information to population factors alone. Specifically, the c-statistic was improved from 0.62 (null model 95% CI=[0.60, 0.65]) to 0.67 (full model 95% CI=[0.65, 0.69]), with a p-value of=4.583 × 10^–6^. These results were also replicated across both stratified sub-cohorts of British Bangladeshi (Full model AUC=0.66, 95% CI=[0.63–0.69] vs null model AUC=0.60, 95% CI=[0.58–0.63]) and Pakistani women (Full model AUC=0.75, 95% CI=[0.71–0.80] vs null model AUC=0.71, 95% CI=[0.67–0.76], *P*=.0088).

Zöllner. Understanding the potential contribution of polygenic risk scores to the prediction of gestational and type 2 diabetes in women from British Pakistani and Bangladeshi groups. Am J Obstet Gynecol MFM 2025.

## Comment

### Principal findings

In this study of 10,931 women of South Asian ethnic origin, the addition of genetic information to epidemiological risk factors offered some improvement to the prediction of both primary gestational diabetes, and the subsequent development of type 2 diabetes following GDM. By combining epidemiological and genetic risk factors, it is possible to identify women at a substantially higher risk than background (30%) of developing type 2 diabetes following GDM, offering a possible cohort for therapeutic intervention. While the improvement in predictive power may appear incremental, this approach mirrors the utility of PRS in other conditions, such as cardiovascular disease, where PRS has been shown to significantly enhance risk stratification and identify actionable high-risk groups.^20^ Furthermore, evidence from the RADIEL study indicates that genetic predisposition to diabetes modifies the response to lifestyle interventions aimed at preventing GDM and postpartum diabetes. This suggests that PRS could enable tailoring of interventions, providing an opportunity to optimize outcomes for those at the greatest genetic risk. Such integration has the potential to enable precision medicine, offering targeted follow-up and intervention for those at greatest risk.^21^

### Results in the context of what is known

It has previously been demonstrated that incorporating genetic information can improve the prediction of type 2 diabetes after GDM.^5^ However, the potential contribution of genetics has not been fully explained. In our cohort, we were able to stratify women based on their genetic risk, with women in the highest risk group at 1.6 times the risk of the group overall (30% compared to 19%) and women in the lowest risk group at half the risk (10% compared to 19%).

The underlying etiology of type 2 diabetes is multifactorial, including genetic, epigenetic, environmental, and nutritional factors. Both type 2 and gestational diabetes are diagnoses subject to changing definition, with the threshold of diagnosis for each coming down in recent years,^22^ based on heterogenous evidence with different endpoints in mind and on different diagnostic strategies. The two pathologies are not exactly analogous and there remain outstanding questions about both underlying genotypes and phenotypes of both. This study indicates that, in this South Asian cohort, underlying genetic susceptibility has the potential to clarify this understanding.

### Strengths and limitations

This study's main strength lies in the data source: this is a large population-based study of women at high underlying risk of gestational and type 2 diabetes with linked health and genomic data to improve the prediction of diabetes.

This study is limited by the relative homogeneity of the cohort, potentially limiting translation into wider populations; further analysis and external validation will be required to understand the implications for women from other ethnic groups. This study is further limited by data availability, including the need to impute data missing in EHRs. It is possible that additional clinical information may offer further improvement of prediction.

Although women with GDM in our study were statistically significantly older and had a higher BMI, these differences may not be clinically significant and should be interpreted cautiously. Similarly, the observed differences in parity status were influenced by our methodological decision to align with other published studies by selecting younger women with GDM and older controls.^10^

### Clinical and research implications

This study demonstrates the feasibility of using genetic information to identify women at higher risk of type 2 diabetes after GDM. Identifying women at very high risk for ongoing diabetes after gestational diabetes would provide a cohort for investigation of ongoing intervention in the immediate postnatal period and prioritization for more intensive postnatal monitoring, to improve outcomes both for future pregnancies and lifelong. This will require additional development and external validation.

This study also demonstrates the small potential use of genetic information to improve prediction of gestational diabetes in this high-risk cohort. In practice, women from South Asian ethnic groups are universally recommended to have screening for gestational diabetes at 24 to 28 weeks of gestation in high-income settings^15^^,^^23^ and, given the severity of a missed diagnosis of gestational diabetes, this is unlikely to be altered unless genetic information could be used to rule-out gestational diabetes. This suggests that genetic studies in gestational diabetes should not be focused on identification in this cohort but rather on understanding disease trajectory, treatment strategies, and future risk, including recurrence in women planning subsequent pregnancy.

## Conclusions

This study, in a large cohort of women from British Bangladeshi and Pakistani backgrounds, demonstrates the feasibility and potential utility of genetic information combined with epidemiological risk factors to identify women at high risk of type 2 diabetes after gestational diabetes. Further research should focus on the development of risk prediction tools and evaluation of opportunities for intervention in higher-risk cohorts.

## CRediT authorship contribution statement

**Julia Zöllner:** Writing – review & editing, Writing – original draft, Methodology, Formal analysis, Data curation, Conceptualization. **Binur Orazumbekova:** Methodology, Formal analysis. **Sam Hodgson:** Writing – review & editing, Methodology, Formal analysis. **David A. van Heel:** Writing – review & editing, Supervision. **Stamatina Iliodromiti:** Writing – review & editing, Supervision. **Moneeza Siddiqui:** Writing – review & editing, Methodology, Conceptualization. **Rohini Mathur:** Writing – review & editing, Supervision, Investigation. **Sarah Finer:** Writing – review & editing, Supervision, Project administration, Data curation, Conceptualization. **Jennifer Jardine:** Writing – review & editing, Writing – original draft, Methodology, Data curation.

## Appendix Supplementary materials

Supplementary Methods. Description of quality control, imputation of genotype data, as well as filtering and principal component analysis. Image, application 1

Supplementary Table 1. Table of included SNOMED or ICD-10 codes Image, application 2

Supplementary Figure 1. Comparison of different PRS performance with predicting GDM (beta >0.3). GDM, gestational diabetes; PRS, polygenic risk score. Image, image 3

Supplementary Figure 2. Distribution of PRS (*z*-score) by GDM status. GDM, gestational diabetes; PRS, polygenic risk score. Image, image 4

Supplementary Figure 3. Risk of developing GDM by PRS decile divided by different genetic ancestry. GDM, gestational diabetes; PRS, polygenic risk score. Image, image 5

Supplementary Figure 4. Progression (days) to type 2 diabetes between top and bottom deciles. Image, image 6

Supplementary Figure 5. Age distribution among bottom and top decile of PRS. PRS, polygenic risk score. Image, image 7

## Footnotes

Supplementary material associated with this article can be found in the online version at doi:10.1016/j.xagr.2025.100457.
