# Supplementary figures and images for: Understanding the potential contribution of polygenic risk scores to the prediction of gestational and type 2 diabetes in women from British Pakistani and Bangladeshi groups: a cohort study in Genes and Health
Source: AJOG Glob Rep. 2025 Feb 21;5(2):100457. doi: 10.1016/j.xagr.2025.100457 (PMC11976246; doi:10.1016/j.xagr.2025.100457)

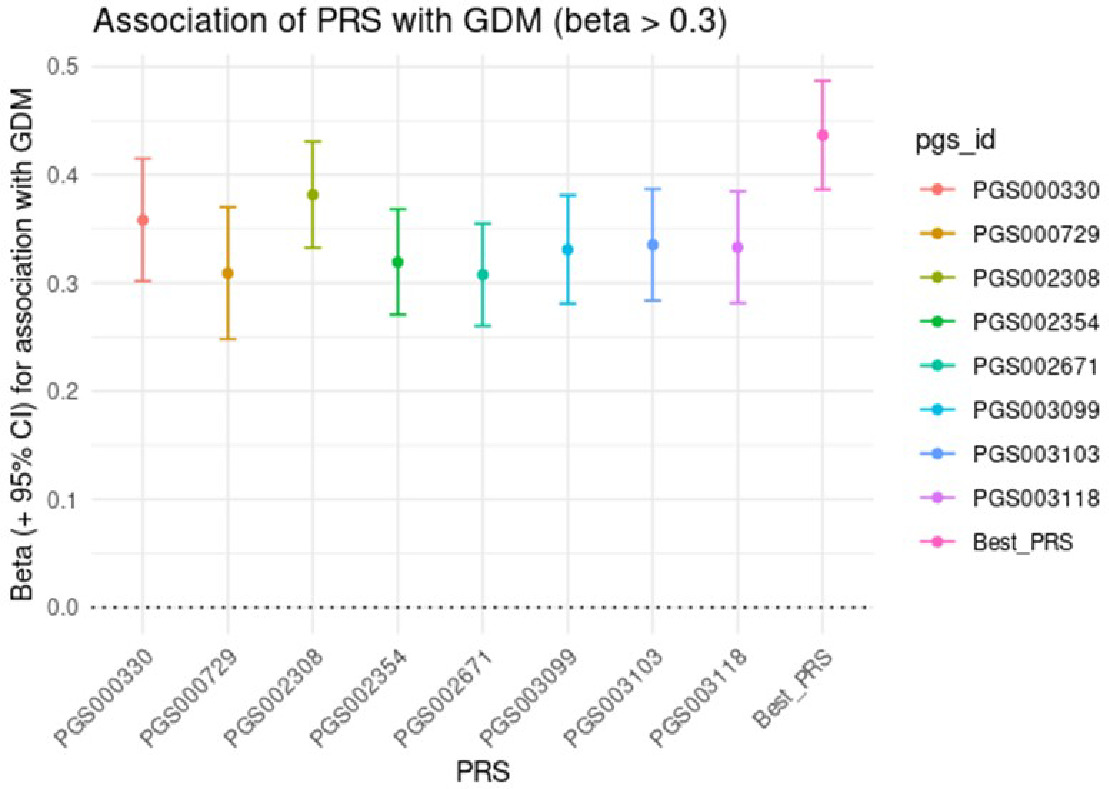

Supplement: Supplementary file 3 — Supplementary Figure 1. Comparison of different PRS performance with predicting GDM (beta >0.3). GDM, gestational diabetes; PRS, polygenic risk score. [file mmc3.jpg]

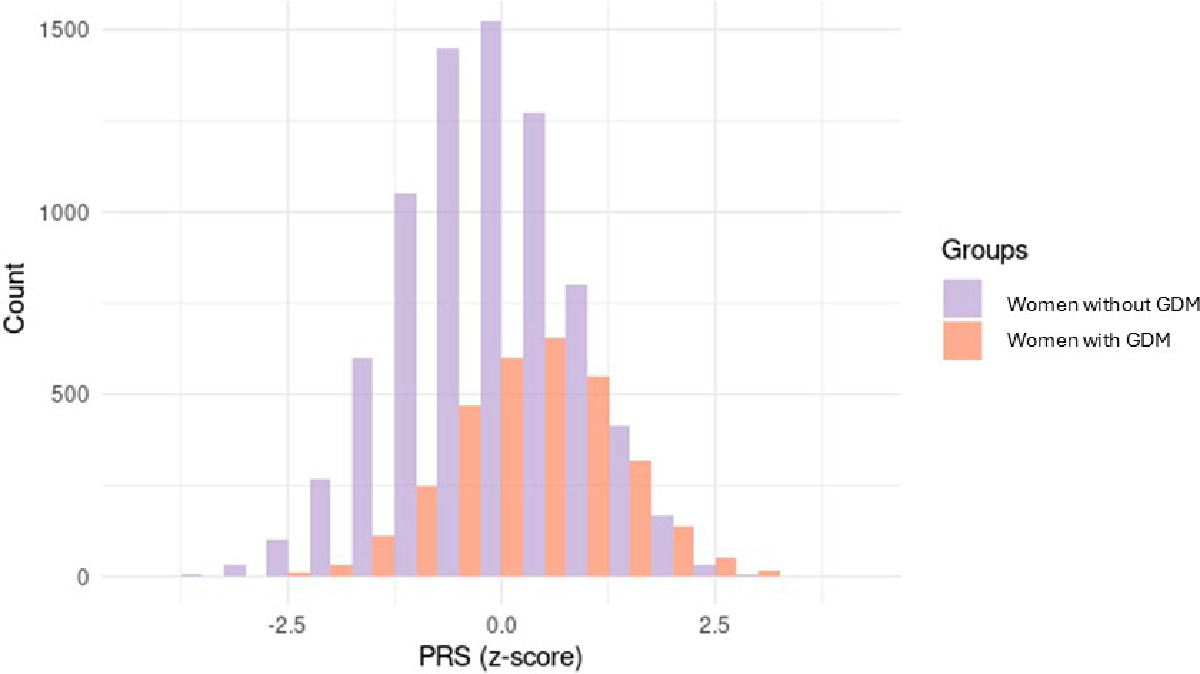

Supplement: Supplementary file 4 — Supplementary Figure 2. Distribution of PRS (z-score) by GDM status. GDM, gestational diabetes; PRS, polygenic risk score. [file mmc4.jpg]

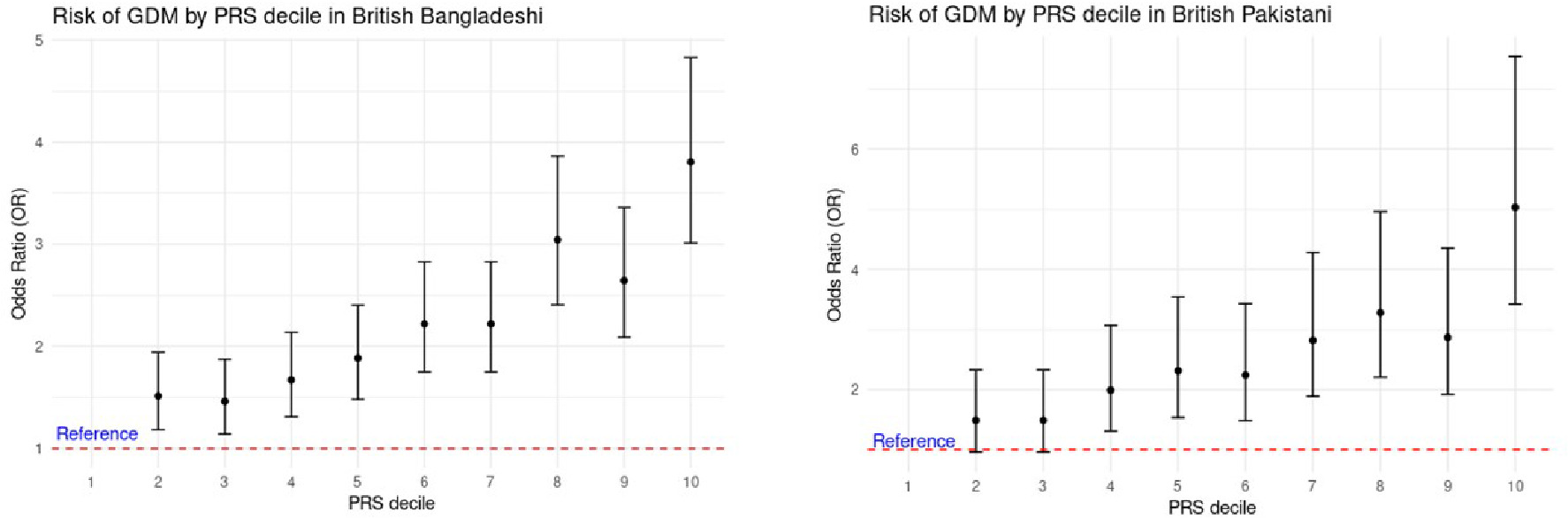

Supplement: Supplementary file 5 — Supplementary Figure 3. Risk of developing GDM by PRS decile divided by different genetic ancestry. GDM, gestational diabetes; PRS, polygenic risk score. [file mmc5.jpg]

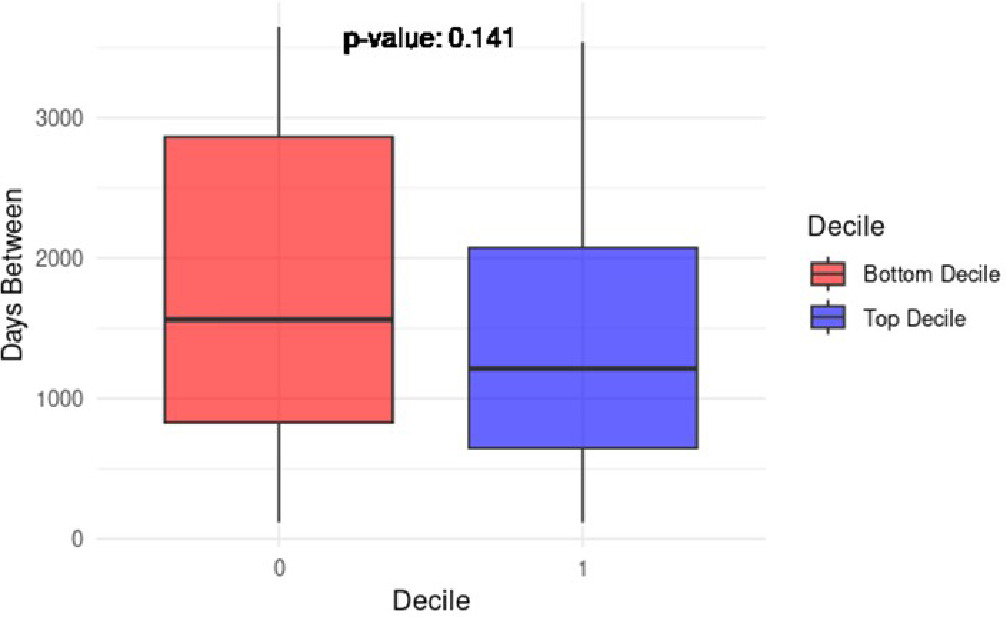

Supplement: Supplementary file 6 — Supplementary Figure 4. Progression (days) to type 2 diabetes between top and bottom deciles. [file mmc6.jpg]

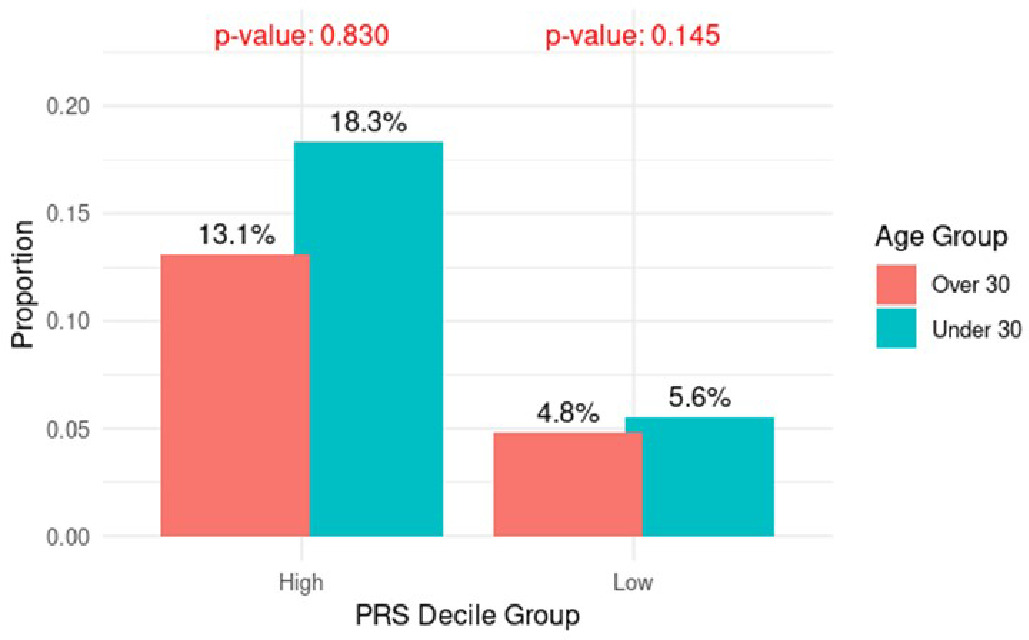

Supplement: Supplementary file 7 — Supplementary Figure 5. Age distribution among bottom and top decile of PRS. PRS, polygenic risk score. [file mmc7.jpg]
